# Improving fever management of hospitalized children with cancer: barriers, facilitators, and proposed interventions from healthcare providers in Kenya

**DOI:** 10.3389/fonc.2025.1620316

**Published:** 2025-09-09

**Authors:** Everlyn Kisembe, C. Nathan Nessle, Julia Dettinger, Lenah Nyamusi, Sarah Kinja, Mercy Ndung’u, Sandra Langat, Kenneth Busby, Gilbert Olbara, Terry A. Vik, Cheryl A. Moyer, Festus Njuguna

**Affiliations:** ^1^ School of Arts and Social Sciences, Moi University, Eldoret, Kenya; ^2^ Behavioral and Social Science Research Working Group, Academic Model for Providing Access to Healthcare (AMPATH), Moi University, Eldoret, Kenya; ^3^ Department of Pediatrics, University of Michigan, Ann Arbor, MI, United States; ^4^ Division of Pediatric Hematology Oncology, University of Michigan, Ann Arbor, MI, United States; ^5^ Fogarty International Center, National Institute of Health, Bethesda, MD, United States; ^6^ Department of Global Health, University of Washington, Seattle, WA, United States; ^7^ Academic Model for Providing Access to Healthcare, Eldoret, Kenya; ^8^ Emma Children’s Hospital of the Amsterdam UMC, Vrije Universiteit, Amsterdam, Netherlands; ^9^ Department Pediatrics, Division Hematology-Oncology, University of North Carolina at Chapel Hill, Chapel Hill, NC, United States; ^10^ Department of Child Health and Pediatrics, Moi Teaching and Referral Hospital, Eldoret, Kenya; ^11^ Department of Pediatrics, Division of Hematology-Oncology, Riley Hospital for Children, Indiana University School of Medicine, Indianapolis, IN, United States; ^12^ Department of Learning Health Sciences, University of Michigan Medical School, Ann Arbor, MI, United States; ^13^ Department of Child Health and Pediatrics, Moi University, Eldoret, Kenya

**Keywords:** febrile neutropenia, pediatric oncology, antibiotic, blood stream infection, guideline, implementation science

## Abstract

Children with cancer in lower-middle-income countries (LMICs) are at increased risk of dying from infections. Prompt treatment of fever episodes improves outcomes, yet poorly described challenges impair management. This qualitative study explored healthcare provider perspectives on barriers to and facilitators of inpatient fever management in children with cancer at a public tertiary referral children’s hospital in Kenya. Healthcare providers involved in fever management were recruited. Semi-structured interviews were audio-recorded, transcribed verbatim, and entered into NVivo software. Coding was informed by a theoretical fever framework and the Consolidated Framework for Implementation Research. Thematic analysis and mind mapping identified recurrent themes and subthemes. Strategies were mapped to identified barriers. The sixteen participants included nurses (n = 2), clinicians (n = 6), pharmacists (n = 2), phlebotomists (n = 2), and microbiology laboratory staff (n = 4). We identified three overarching themes: empowerment of healthcare providers and caregivers, the importance of timely management, and teamwork/human resource availability. Healthcare provider attributes served as facilitators: motivation to improve care, eagerness to learn, willingness to change practice, and need for treatment guidance. Factors within the hospital system were barriers, with subthemes including poor communication between cadres, delays in laboratory results, and staffing shortages. Participants suggested knowledge sharing, a treatment guideline, task shifting, and hiring additional healthcare providers as potential interventions. Managing fever episodes in children with cancer is complex, requiring multiple cadres of healthcare providers and caregiver participation. The proposed interventions may overcome barriers, but future studies are needed to assess the effectiveness of these strategies in improving fever management.

## Introduction

Children with cancer in lower-middle-income countries (LMICs) are 20–30 times more likely to die from fever episodes and infections (1–3), which is likely multifactorial in etiology (1, 3, 4). A high incidence of bloodstream infections, exceeding 28% (5), with an increased prevalence of multidrug-resistant organisms has been reported (6, 7). Children with cancer in LMICs frequently present with severe illness at the time of infection (1, 3), with up to 55% requiring intensive care (3). Staffing shortages are commonplace and low nurse-to-patient ratios contribute to delays, worse outcomes (8), and higher sepsis-related mortality (9). Many pediatric oncologists in LMICs employ reduced-intensity treatment regimens to mitigate the increased infection and treatment-related mortality. While this approach improves outcomes in LMIC settings, survival remains lower compared with high-income country (HIC) settings (10, 11). Improving infection management—a leading cause of death in children with cancer in LMICs (12),—not only benefits those with infections but also brings oncologists in LMICs closer to safely implementing conventional regimens (11, 12).

Effective fever management in children with cancer—that is, the early detection of severe illness and prompt management with blood cultures and antibiotics—remains challenging at LMIC centers (13, 14). The risk of infection-related mortality increases with moderate (>3 h) and severe (>24 h) treatment delays (1, 15, 16). A multicenter study in Africa revealed that few antibiotics were administered within 3 h of fever detection, almost half were administered after >24 h, and nearly all blood cultures were drawn after antibiotics (1). Early detection and management of fever episodes can reduce cancer mortality risk (17, 18), decrease adverse events (19), lower intensive care unit admissions (20), reduce sepsis-related mortality (15, 18, 20), and improve overall outcomes in pediatric oncology fever episodes in LMICs (4).

**Figure 1:**
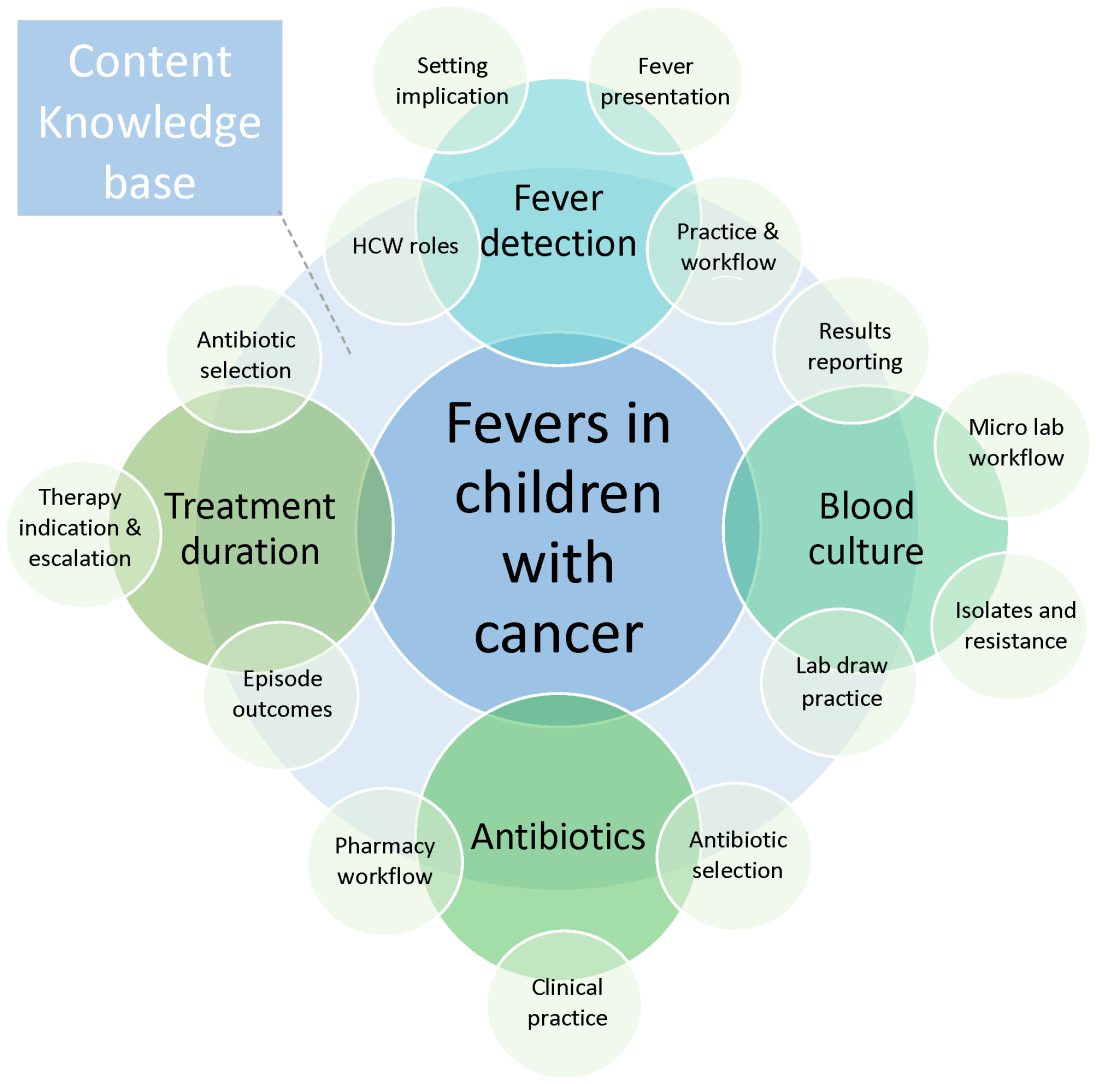
Fever theoretical framework developed during the study planning stage to ensure a comprehensive approach in data collection, describing the main areas of the fever management process (fever detection, blood cultures, antibiotics, and treatment duration). Each area has distinct aspects to consider, denoted by the small light green circles overlapping in the respective areas. HCW, healthcare worker.

Despite the known poor outcomes associated with fever episodes, improving fever management in children with cancer in LMICs is complex. Improved understanding of the barriers and facilitators of a sepsis screening tool in LMICs (21) enabled researchers to identify strategies (22) that enhanced implementation and reduced mortality (23). Conducting a needs assessment that includes the description of barriers and facilitators is a valuable first step before implementing an evidence-based intervention (24–27). However, the barriers and facilitators of timely fever management in children with cancer in LMICs are not well described. Our team applied the Consolidated Framework for Implementation Research (CFIR) (28) and developed the Fever Theoretical Framework (29) (**Figure 1**) to comprehensively describe fever management, which facilitated the identification of strategies and interventions for future quality improvement initiatives in our large, tertiary public referral hospital in Kenya.

## Methods

### Study design

This phenomenological qualitative study was part of a larger convergent mixed-methods study (29) conducted at Moi Teaching and Referral Hospital (MTRH) in Eldoret, Kenya. The study team included doctoral-trained qualitative researchers (EK, CM); a doctoral-trained implementation scientist (JD); trained qualitative researchers (LN, MN, SK, CNN); and pediatric oncologists and researchers (CNN, KB, TAV, FN, GO), all with experience in the Kenyan healthcare system. The Fever Theoretical Framework (29) was developed by this team to guide the comprehensive evaluation of diagnostic and treatment workflows for fevers in hospitalized children with cancer at MTRH. We also used the Consolidated Framework for Implementation Research (CFIR (24) to guide the evaluation of implementation barriers within the hospital setting. This report followed the Standards for Reporting Qualitative Research (SRQR) where applicable (30) (Appendix 1).

### Study setting

Moi Teaching and Referral Hospital (MTRH) is the largest government referral hospital in western Kenya, serving a population of more than 24 million (31). It has strong research capacity through a longstanding partnership with a global consortium of academic universities (32). Children receive care at Shoe4Africa, the largest public children’s hospital in East Africa, which is a 250-bed facility within the MTRH system and includes five beds in the pediatric intensive care unit. The pediatric oncology program manages more than 300 new childhood cancer diagnoses annually (33). The clinical microbiology laboratory has state-of-the-art capabilities to identify bacterial isolates, determine antibiotic susceptibilities, and perform genetic tuberculosis testing. Fever management in children with cancer was adapted from society recommendations, including empiric broad-spectrum antibiotic administration and an infectious disease diagnostic evaluation based on clinical presentation (34). Sepsis screening had not been implemented into routine clinical practice.

### Interview participants

Eligible participants were healthcare providers who delivered direct or indirect care for hospitalized pediatric cancer patients with fevers at MTRH. Demographic data were not collected to prevent participant identification. Participants were approached in person by a member of the research team during recruitment activities. Purposive and snowball sampling techniques were used during the 3-month recruitment period (15 March to 30 June 2023) to ensure a wide range of experiences, roles, and job descriptions were represented. Sixteen participants were recruited: nurses (n = 2), pediatric oncology clinicians (n = 6), pharmacists (n = 2), phlebotomists (n = 2), and microbiology laboratory staff (n = 4). Written informed consent was obtained. No financial incentives or reimbursements were provided.

### Qualitative data collection: semi-structured interview

The semi-structured interview guide (Appendix 2) was informed by the Fever Theoretical Framework (29) and CFIR 2.0 (28), tailored to the local context, and piloted before data collection. Field notes were documented during the interview process to inform data analysis. A total of 16 interviews were conducted in person in English by CNN and LN and continued until thematic saturation (35) was reached—that is, the point at which additional interviews did not yield new insights, as determined by rapid analysis. Interviews were digitally recorded, de-identified, transcribed verbatim (MN, CNN), entered into NVivo 12.0 for coding, and reviewed (MN, SK, CNN, EK).

### Qualitative thematic analysis

Three team members (SK, CNN, EK) first familiarized themselves with the transcript data and the interview guide, then read every sentence and inductively coded relevant segments of data related to fever management in children with cancer to develop the initial codebook. This was done through three transcripts, informed by the study frameworks (28, 29) and the interview guide (Appendix 3). As additional transcripts were analyzed, the codebook was updated to capture new patterns, refine existing themes, and ensure consistency. Changes included adding new codes, adjusting categories, and merging similar themes to improve the analysis. Thematic analysis was conducted (CNN, EK, CM, FN) using mind mapping (36, 37) and the Attride-Sterling method (38) until consensus themes and subthemes were identified through iterative meetings held from 1 July 2023 to June 2024. Mind maps are diagrams that can be an effective method to communicate a comprehensive understanding of key concepts within a subject matter (36, 37). The Attride-Sterling method followed six iterative steps: coding material, identifying themes, constructing thematic networks, exploring thematic networks, summarizing thematic networks, and interpreting patterns. The diverse perspectives of the analysis team, data triangulation, and sharing of insights during meetings supported the rigor of the analysis. Finally, all investigators selected and agreed on vivid de-identified quotes to illustrate the described themes.

### Strategy mapping

Informed by the study’s Fever Theoretical Framework (29) and the methodological steps of Fernandez et al. (26), we (CNN, JD, FN) informally mapped (26) expert recommendations for implementing change (ERIC) strategies (39, 40) and behavior change techniques (41) to the recurrent interventions suggested by participants to mitigate identified barriers. Participants were asked to provide solutions to the challenges they described multiple times during the interview process (Appendix 3). During the mapping process, we iteratively revisited the barriers and facilitators based on CFIR domains and the Fever Theoretical Framework to strengthen our understanding of the proposed interventions and strategies. We then selected implementation strategies that best aligned with the proposed solutions, taking into account perceived barriers and facilitators to improve uptake (26, 39, 40). For example, inconsistent antibiotic practice was identified as a barrier, the proposed facilitator was the adoption of a protocol, and the most suitable implementation strategy was a standardized antibiotic treatment protocol communicated through a credible source. Strategies were then arranged by barrier level and briefly described in relation to that barrier. The thematic results and strategy mapping were reflexively reported and shared with the interview participants and other healthcare providers across cadres in a collaborative, multidisciplinary small-group meeting led by FN, CNN, and LN. The goal of this study was to enhance understanding of the barriers and facilitators to fever management, with the aim of informing implementation strategies that address the challenges faced by healthcare providers (29). Therefore, some formal tasks of the implementation mapping process—such as evaluating implementation outcomes, protocol development, and identifying outcomes and objectives (26)—were beyond the scope of this report.

### Ethics and dissemination

The study received ethical approval from the institutional review boards of the University of Michigan (HUM0225674), MTRH (0004273), and the MTRH chief executive officer. The study was registered with the National Commission for Science, Technology, and Innovation (P/23/22885).

## Results

We recruited a heterogeneous group of 16 healthcare providers: bedside and charge nurses, clinical officers, pediatric oncology medical officers, consultant pediatric oncologists, phlebotomists, pediatric oncology clinical pharmacists, an antimicrobial clinical pharmacist, pharmacy technicians, microbiology laboratory technicians, microbiology laboratory management, and doctoral clinical microbiologists. Participants described multiple barriers, facilitators, and proposed interventions to improve management. The average interview length was 55 min (range: 24–111 min). We identified recurrent themes and organized the barriers, facilitators, and interventions under the most appropriate thematic categories, though the complexities of fever management in children with cancer were evident. **Table 1** includes supportive quotations from participants for major themes and subthemes. Participants readily recalled memorable scenarios where multiple challenges in fever management—such as lack of standardization, delayed results reporting, poor communication, and delays in detection and management—resulted in poor outcomes for a child:

**Table 1:** Representative quotes from participants regarding fever management in hospitalized Kenyan children with cancer.

Theme	Barriers	Facilitators	Interventions
Importance of timely management			
	***Fever management processes***	***Eagerness to learn***	***Implementation guideline***
	“Since we don’t do just that one specific sample alone, we pool the samples because we have to collect all the samples in that particular ward … but it can sometimes delay to 4 hours because they are being pooled for transportation to the processing lab…” - Phlebotomist 2 “…someone will get a fever at a point when the doctor is not around and then, maybe they don’t know what to do, or they are overwhelmed by work and they will not notice this fever, and they had something else to do.” - Charge Nurse 1	“We don’t get … so many in-job trainings, especially on a small area of management like Fever … very hard to find even in, in conferences, it’s very hard to find.” - Pediatric Oncologist 2 “We only attend the online CMEs to update us. But for physical trainings, it has been long. Unless the one we just organized within the department or within the unit.” Pharmacy Technician 1	“So I think it is just us as clinicians to implement the protocols which are existing or which are coming up because that is where we don’t really do our best.” - Clinical Officer 1 “Would be best to have a protocol. Like if the child has this, do this, like a guideline … for fevers so that it’s clear and it’s standardized for all that.” - Clinical Pharmacist 1
		***Innovative, creative solutions***	***Effective computer system***
		“Research like this already, it has so many things. If you have other people who are doing research of course they can bring in more personnel.” - Clinical Officer 1	“I do wish that in the near future all the results will be integrated into the system … where the doctor just clicks the button and sees all the results.” - Phlebotomist 2
Teamwork and human resource availability			
	***Delays in results reporting***	***Multidisciplinary team valued***	***Add healthcare worker personnel***
	“Secondly, is getting these results in a timely manner. would be a bit more useful … but it’s not actually helping us do the intervention.” - Pediatric Oncologist 2 “We had delays in lab works making treatment of the patients quite hard. So, prescribing antibiotics is quite a challenge to some children.” - Clinical Officer 3	“It’s a multi-disciplinary team and everyone has a role to play in the management of these children.” - Charge Nurse 1 “I will say it’s the human resource. It’s us. I would probably say, we are the ones who are making things better. Just having, I have a really good team. It’s amazing, the nurses are amazing. We work with what we have. My consultants are amazing. My clinical officers are amazing, my parents, once we become friends. Initially sometimes there is some resistance but, I feel like it’s the human resource that is working, because as much as these things, these small things, are not there, when you have a good team behind you which tries.” - Medical Officer 1	“…I would wish we have more staffing so that when the patients spike fever if it’s an infection then it is managed accordingly.” - Phlebotomist 2 “I would hire so many people. I would hire the adequate number or the ideal number of recommended personnel.” - Medical Officer 1
	***High workload, low staffing***		***Facilitate communication***
	“No, we don’t work at night … weekends. The doctor on duty will … pick the sample but the problem is microbiology is normally closed at night.” - Phlebotomist 1		“For now, if they can’t post, they can call us. Maybe something which needs to be acted upon immediately.” - Clinical Officer 2 “We are now in the smart technology. Even developing. Just a smart, something short that is able to be inbuilt in the hospital system. That when a fever is recorded, you actually—it’s able to be flagged.” - Clinical Officer 1
	***Poor communication between cadres***		
	“There’s really some miscommunication between the lab and the ward … you get the results … and the clinicians have not seen it.” - Clinical Pharmacist 1 “…we inform the doctor. But that’s not the case. Because of the work load…” - Microbiologist Lab Director 1		
Empowerment of healthcare providers and caregivers			
	***Knowledge gap in task shifting***	***Motivated to deliver excellent care***	***Task shifting***
	“These parents move from very well, a very limited a low level of education … to very educated mothers who understand everything and actually can actually notice or actually notify you what’s the healthcare providers are doing wrong.” - Pediatric Oncologist 2 “And then the knowledge gap. I think knowledge gap is a big one. And then I think for most of the cases it should be … The knowledge gap.” - Clinical Pharmacist 1	“Yes. As soon as somebody is said to have a fever, then we start monitoring that patient closely. We might do two hourly, three hourly or four hourly temperature check. We do the lab works the same day, and if they have another spike within the four hours, then we definitely start antibiotic treatment for them. But then you have to rule out other things.” - Clinical Officer 3	“Yeah, [students] help us, uh, especially in transporting of samples. It makes our work very easy when they’re around.” - Phlebotomist 1 “…when that caregiver is there … especially when like now when the nursing team is overwhelmed … at least we can see a smooth running because (the caregiver) is able to tell us in due time.” - Charge Nurse 2
		***High primary role knowledge***	***Education, knowledge sharing***
		“Right now, it’s the phlebotomists who are taking blood from the patients and also they are trained on how to … take special attention to blood cultures because of contamination, to avoid contamination.” - Microbiologist Lab Director 1	“We need to empower the clinicians … to have adequate knowledge on responding to fever at the right time. The other thing … is empowering the laboratory team. Because from what I see, either there is delay in— we just need to see a way of making a synergetic effect. That we have all these things happening at the right time.” - Clinical Officer 1

“… it’s like we delayed starting antibiotics because we were debating what to give and what to start, because we were waiting for the blood works to come out. Yes, so we were waiting for a hemogram to see what is happening. We took some different samples for different investigations for malaria. Surprisingly it came out to be a neutropenic fever and we didn’t intervene early enough. It was so sad losing that patient.” - Clinical Officer 2

### Importance of timely management

Many participants recognized that fever and neutropenia in children with cancer constitute an oncologic emergency associated with poor outcomes, and the importance of timely management was frequently emphasized. Participants described several barriers that prevented prompt management:

“[Fever management is] not done in a [timely] manner. It’s most of the time, I could say up to 70 to 80% is not done in a timely manner. There are some lags that we have a lot because of how the system works, basically.” - Pediatric Oncologist 2

Participants identified challenges across each step of the diagnostic and management continuum—from the accurate detection of a fever episode, to requesting and obtaining a blood culture, ordering and administering an antibiotic, and delays in microbiology results reporting due to the absence of an electronic system:

“The thermal gun [non-contact infrared thermometer] can get this misleading fever … we can take the temperatures but it’s still low and the child is ill, yes.” - Pediatric Oncologist 1

“We were with I.T [information technology] … and they were speaking of one month [to start the electronic system]. But that is the same thing that happened last year.” -Microbiologist Lab Director 1

Several facilitators also fit within this theme. Many participants recommended innovative, creative solutions to the challenges and expressed eagerness to learn how to improve their clinical practice, emphasizing their desire for more training:

“We do have seminars and webinars … exchange ideas with other institutions, other clinicians, other specialties and consultants … We do have trainings very frequently, several times in a year.” -Medical Officer 1

Participants further suggested that successful implementation of a fever treatment guideline and an effective electronic system would substantially improve fever management:

“I think the one single [best] way of doing this … is having protocols, having protocols or job aids for the people. this is how we are going to approach fevers.” - Clinical Pharmacist 1

“…most of those things will be sorted out by the system if it takes effect properly as designed.” -Clinical Officer 1

At least one participant felt that current fever management occurred quickly: “A nurse will come from the ward to pick that drug immediately.” (Pharmacy Technician 1).

### Teamwork and human resource availability

Nearly all participants recognized that a multidisciplinary team is valuable in fever management. However, barriers within the hospital system setting limited teamwork effectiveness and led to poor practice habits. When asked to describe a valued aspect of fever management, participants frequently described the team:

“…[the] multidisciplinary team who are involved in fever management. That is the nurses, the clinicians and also the clinical pharmacists … So I think that has really strengthened our intervention in terms of fever management.” - Charge Nurse 2

High workloads faced by healthcare providers due to staffing shortages subsequently caused delays in results reporting and poor communication between cadres, which negatively impacted teamwork:

“The nurses really, I mean like now there is a shortage. We only have three nurses *for 64 patients* [added emphasis], so that becomes very difficult especially when to do vitals.” - Medical Officer 1

Participants suggested several interventions, such as increasing healthcare personnel and effective facilitation of communication, to enhance teamwork, practice habits, and human resource availability:

“What else can help to also maybe have more clinician at night because it’s usually very few and there’s nothing we can do about the time for rounds [when clinicians must be present due to the workload] because it’s different rounds [at night].” - Clinical Pharmacist 1

“We need to keep talking. Like I said the way the human brains work, once it is used to do things in a particular manner it needs several reminders to change practice.” - Pediatric Oncologist 1

### Empowerment of healthcare providers and caregivers

The third recurrent theme was the empowerment of healthcare providers and caregivers. Participants noted that while task shifting from overburdened providers, such as phlebotomists or nurses, to trainees or caregivers may be an ideal intervention, knowledge gaps may exist during task shifting:

“… the nurse is allocating some students to do the vitals … at times cannot give you good information, unless the parent now comes and says ‘my child is feverish, my child is feeling hot’. It’s where you tell the qualified sister nurse, ‘this child is hot, and can we check the temperatures?’ Then you find a very different reading.” - Clinical Officer 2

Despite the challenges faced by healthcare providers involved in the management of fevers in children with cancer, participants consistently demonstrated strong knowledge and eagerness to deliver excellent care:

“[Fever] is constantly given a priority because it’s an oncological emergency … if you don’t respond at the right time, definitely, you will lose that child.” - Clinical Officer 1

“It’s us who make the system work because the system is really confined … So having that team work … working together to try and help you with as little resources that we have, that is the thing that is pushing us forward. Yeah.” - Medical Officer 1

### Intricacies of fever management

**Figure 2:**
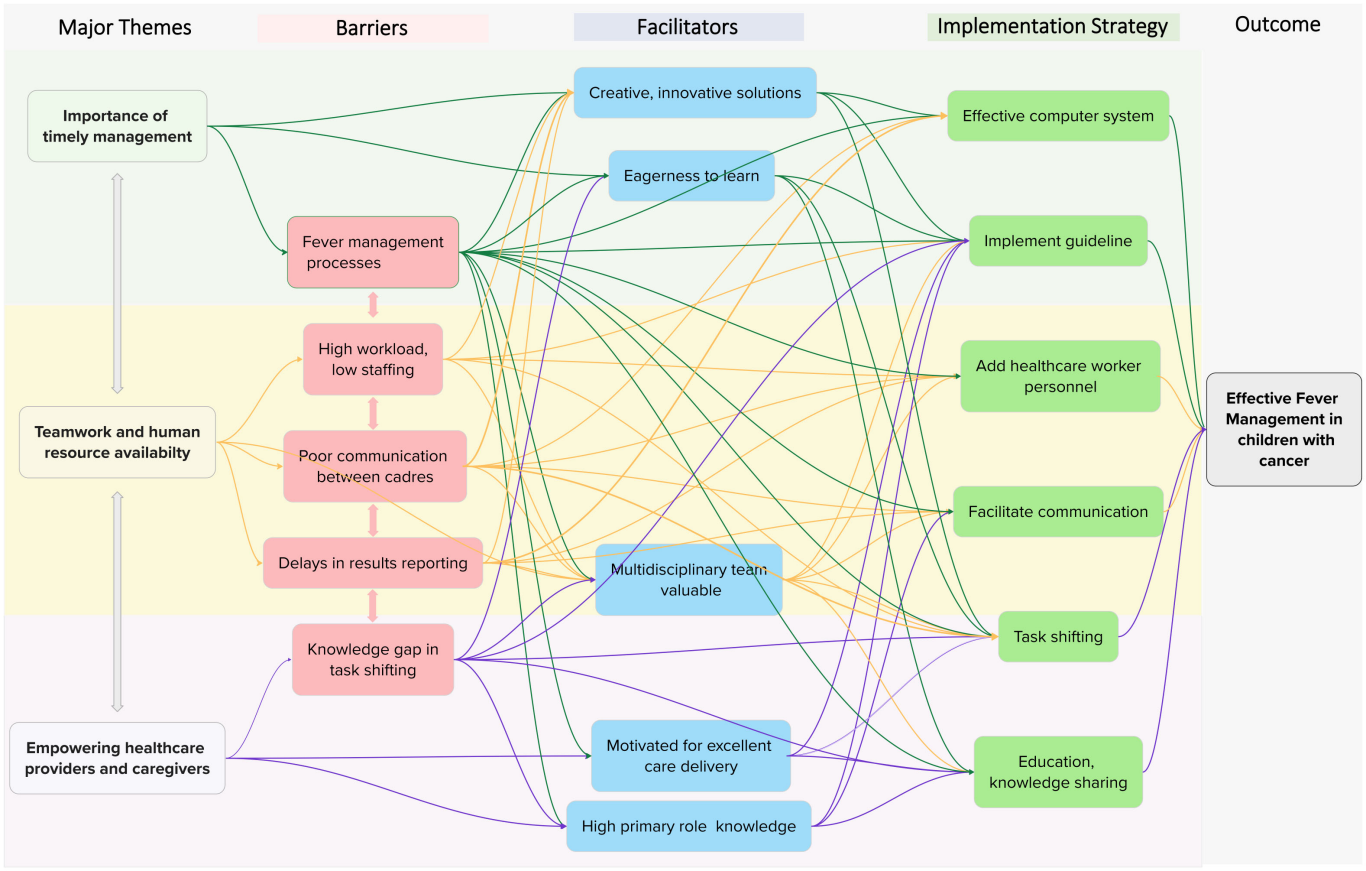
Mind map of the thematic analysis. The mind mapping exercise began following consensus identification of themes and subthemes, at which point we recognized complex relationships between the initial thematic results that were not previously captured. The color scheme is as follows: green = “importance of timely management”; yellow = “teamwork and human resource availability”; purple = “empowering healthcare providers and caregivers.” Subthemes are color-coded by category: red = barrier, blue = facilitator, green = implementation strategy. Branches connect related themes and subthemes to illustrate the complexity of their relationships.

While we identified three major themes, five barriers, five facilitators, and six interventions, these were interconnected and interacting rather than separate and independent (**Figure 2**). Each barrier was influenced by the others: low staffing led to high workloads; high workloads contributed to delays in blood culture processing and poor communication; and these, in turn, delayed results reporting. Results retrieval on hard copy, when performed during task shifting by a student or healthcare worker who might not appreciate the urgency, further compounded these delays.

Participants described complex relationships between barriers, facilitators, and interventions, with each factor influencing multiple others within the map. For example, the barriers of high workload and low staffing negatively affected fever management processes and the facilitator of a valued multidisciplinary team. These barriers, however, could be mitigated by several interventions and strategies, such as task shifting, guideline implementation, and adding healthcare personnel. Similarly, each strategy addressed multiple barriers and was shaped by several facilitators. For instance, guideline implementation targeted multiple identified barriers (fever management processes, high workload/low staffing, and knowledge gaps in task shifting) and was facilitated by provider attributes such as eagerness to learn, willingness to adopt innovative solutions, valuing a multidisciplinary approach, motivation to deliver excellent care, and strong role-specific knowledge.

### Proposed strategies and interventions

Participants proposed several interventions to comprehensively improve management, summarized in **Figure 2**. **Table 2** describes the ERIC (Expert Recommendations for Implementing Change) implementation strategies (39) and behavior change techniques (41) that were informally mapped to mitigate implementation barriers (26), providing a pragmatic, detailed description of each potential intervention. The most common strategies were use of a credible source, task shifting, use of champions, and facilitating communication. These were organized under the barrier of best fit, although many were applicable across multiple barrier levels.

**Table 2:** Description of strategies and techniques mapped to barriers of inpatient fever management.

Barrier level		Barrier description	Strategy or Technique^*^	Description
**Human resources**	Clinical and laboratory team	Low staff night/weekend	Adding objects, tailoring	Task shifting, hiring more personnel
		Workload limits teamwork	Tailoring	Tailored task shifting, communication
		Delayed fever detection	Credible source, habit formation	Tailored task shifting, device
		Task shifting restrictions	Credible source, tailoring	Tailored task shifting, Champions and manager directives
		Hesitancy to change	Habit formation	Champions and manager directives
		Stay in role	Model change, revise roles	Champions model change
		Roles/responsibility	Revise professional roles	Champions and manager directives
		Task shifting: education	Educational meetings	Routine iterative CMEs in cadres
**Process**	Blood Culture	Delay: ordering	Habit formation	Champions and manager directives, guideline use, communication
		Delay: draw from patient	Local leaders, model change, revise roles, facilitate communication	
		Delay: transport		
		Delay: result reporting		
		Result interpretation	Educational meetings	Routine iterative CMEs in cadres
	Antibiotics	Delay: ordering	Habit formation	Manager directives
		Inconsistent practice	Credible source	Guideline use, CMEs, model change
		Delay: administration	Adding objects, tailoring	Task shifting
		Inaccurate records	Change structure, tailoring	Task shifting; hire more personnel
	Clinical information	Poor communication	Facilitate communication	Use mediums (WhatsApp^®^, order request) to facilitate
**Hospital system**	Hospital Policy	No standardization	Credible source	Guideline use
		Few required education sessions	Educational meetings, institutions	Routine iterative CMEs in cadres
		Restricted work hours: phlebotomy, micro	Adding objects, tailoring	Task shifting; hire more personnel
	Hospital structure	Poor hygiene	Change structure, education	Cleaning, hand hygiene
		Malnutrition	Tailoring	Nutritional management
		Overcrowding	Change structure, credible source	LOS policy, isolation room, leverage outpatient resources
		Electronic medical record not functional	Change record system	Provide feedback to hospital informatics, involve local stakeholders (lab, hospital)
		Results not reported		
		No integration		
		Stock outs	Credible source	Continuous procurement
		Phlebotomy capacity	Change equipment, involve executives	Specialized equipment
		Microbiology lab capacity		Specialized equipment (analysis)

Strategies and techniques mapped to the interventions suggested by interview participants. The barriers are closely related and were arranged according to the best fit, though they may also interact at other barrier levels. Each strategy or technique is mapped to a specific barrier, with a brief description of the potential intervention provided for each. #Expert Recommendation for Implementing Change strategies^33^ *Behavioral Change techniques are associated with a specific action related to a barrier.^34^ LOS, length of stay; CME, continuing medical education; and ICT, information and communication technology.

For example, poor communication between healthcare providers contributed to delays in the reporting of blood culture results. The mapped strategy was “facilitate communication,” and the proposed intervention involved connecting cadres via WhatsApp^®^, a free and widely used medium. However, facilitating communication—along with changing the record system through an electronic medical record—was also considered an appropriate strategy for hospital system–level barriers.

Many of the proposed strategies in **Figure 2** were complex, multilevel interventions likely to require multiple implementation strategies, described in **Table 2**. Guideline implementation, for instance, was viewed as a multilevel intervention capable of addressing several management barriers. Its effective implementation would likely require multiple strategies, outlined in **Table 2**, including iterative educational meetings, facilitating communication, hiring additional personnel, issuing managerial directives, engaging champions, and local tailoring.

## Discussion

To our knowledge, this is the first qualitative research study to provide novel insights into the complex relationships among barriers and facilitators of fever management in children with cancer in a tertiary referral hospital in Kenya, while also describing interventions and strategies to improve management. Most barriers were located within the hospital system (e.g., high workload/low staffing, delays in results reporting), whereas most facilitators of effective fever management were characteristics of healthcare providers (e.g., eagerness to learn, motivation to deliver excellent care). Participants also suggested interventions that addressed multiple barriers (e.g., guideline implementation, facilitating communication). Task shifting from overburdened healthcare providers to patient caregivers emerged as a potentially cost-effective, impactful, yet underutilized strategy to improve management effectiveness. The complexities of fever management highlighted the importance of an effective healthcare system to support care delivery and the value of a multidisciplinary team approach. Pragmatically, this comprehensive description of fever management practices enabled strategy mapping and identification of interventions directed toward barriers to improve clinical practice. This study established a valuable foundation for our team in selecting implementation strategies to improve aspects of fever management, such as blood culture and antibiotic processes.

This study qualitatively expands upon the intricacies of fever management by describing the interactions of barriers, facilitators, and proposed interventions, which largely align with published quantitative reports. Fever management in children with cancer is challenging, and barriers within healthcare systems (e.g., personnel shortages, budget constraints) have been described in LMICs (3, 13). Specifically, nurse shortages contribute to high workloads and lead to delays in fever detection and management (42). Our thematic findings also support quantitative reports of delays in blood culture and antibiotic processes (1, 14), although antibiotic resistance or shortages were not described as barriers in this study (2, 3, 5, 6). Other LMIC centers have highlighted the need to develop fever treatment guidelines, noted challenges to guideline implementation, and recognized the importance of strategies to support guideline implementation (13, 14). Given the described challenges around guideline implementation (13, 14) and the intricate barriers identified here, the use of multiple implementation strategies (e.g., educational activities, facilitating communication) may improve uptake of evidence-based interventions that enhance fever management. Fever and neutropenia have been described as an oncologic emergency associated with death (1, 3, 4). However, early detection and management of sepsis did not appear as either a barrier or facilitator in our study (21, 23). Poor knowledge during task shifting, especially to trainees, was described (43), whereas a facilitator of effective management was strong role-specific knowledge (e.g., phlebotomists performing phlebotomy tasks (42). Task shifting, educational activities, knowledge sharing, innovative solutions such as mobile technology (44), and guideline implementation have been previously suggested to improve fever management (13, 14). Importantly, our results connect pragmatic implementation strategies to the complex barriers described by healthcare providers.

As our team reflected on this study, we initially underappreciated the scope of challenges faced when managing fever in children with cancer at our center. We found that mapping these strategies was a valuable exercise for planning future efforts to improve our management.

This study has several strengths. Internal validity was supported by reflexive reporting of results to participants, while external validity was reflected in the concordance of our findings with existing literature. The study intentionally recruited a heterogeneous group of participants, including all cadres of healthcare providers with varying levels of experience in fever management. Additionally, because the study was conducted at the largest tertiary public referral hospital in western Kenya, the results may be generalizable to other similarly resourced public hospitals managing fevers in children with cancer in LMICs. The thematic results reported here aligned with the Fever Theoretical Framework and CFIR (28) used in the study planning. However, as our team continued to use these results to inform subsequent interventions, we noted that explicit inclusion of sepsis detection, septic shock management, and intensive care unit access was absent from the Fever Theoretical Framework—important clinical challenges that became evident after this study.

Despite these strengths, we recognize several limitations. Recruitment in certain cadres was limited due to staffing shortages, although thematic saturation was achieved. Semi-structured interviews inherently pose a risk of response bias, which we attempted to mitigate by establishing relationships with participants and ensuring confidentiality of responses. Additionally, aspects of fever management such as sepsis screening and intensive care unit access did not emerge in the analysis, though they may represent important interventions to consider—possibly reflecting response bias. Although members of the study team shared the thematic results and strategy mapping with interview participants and multiple healthcare providers across represented cadres (nursing, phlebotomy, physician, pharmacy, microbiology laboratory), not every participant was available. As a phenomenological qualitative study, the findings highlight perceptions and experiences, but a future study is needed to determine the impact of the proposed strategies on mitigating barriers to effective fever management—for example, improving blood culture and antibiotic processes, reducing time-to-antibiotics, and facilitating prompt detection of severe illness during a fever episode. Other aspects of fever management, including sepsis screening, antibiotic resistance, and caregiver involvement, should also be considered in future studies.

Our study advances knowledge of the intricacies of fever management in children with cancer in a public hospital in an LMIC. More importantly, we mapped implementation strategies and described interventions to address barriers to effective fever management, many of which are not resource intensive (e.g., use of champions, model change, iterative training, development of a guideline, and WhatsApp^®^ to improve communication). Although our public tertiary referral hospital benefits from an advanced clinical research infrastructure (32), MTRH remains a publicly funded hospital with finite resources, similar to other centers (3, 13, 14). While the management barriers may be generalizable to similarly resourced public tertiary referral hospitals in sub-Saharan Africa, the interventions and strategies should be locally tailored.

A multidisciplinary team and comprehensive approach are likely required to improve fever management of children with cancer; a single strategy (e.g., an effective electronic system or hiring more personnel) would be insufficient to advance care delivery without other supportive strategies and interventions (e.g., facilitating communication, task shifting, and increasing healthcare providers). Our team used these qualitative insights to support the implementation of several multilevel interventions, leveraging many of the implementation strategies directed toward the barriers described by participants. We encourage other pediatric cancer centers to evaluate the applicability of these findings—including barriers, facilitators, strategies, and interventions—within their local settings.

## Conclusion

A multidisciplinary team must navigate barriers within the healthcare system in the management of fevers in children with cancer in LMICs. Given the complexities in managing fevers in children with cancer in LMICs, a multidisciplinary approach to comprehensively improve clinical practice is likely needed to mitigate the intricate multilevel barriers. Attributes of healthcare providers facilitate effective fever management and should be leveraged in the implementation of strategies and interventions to improve care. Hospital system barriers may be mitigated through knowledge sharing, task shifting, guideline implementation, and effective communication. Future studies should measure the implementation and process outcomes of the proposed strategies mapped to these management barriers.

## Data Availability

The original contributions presented in the study are included in the article/supplementary material. Further inquiries can be directed to the corresponding author.
